# Beta Amyloid Deposition Is Not Associated With Cognitive Impairment in Parkinson's Disease

**DOI:** 10.3389/fneur.2019.00391

**Published:** 2019-04-24

**Authors:** Tracy R. Melzer, Megan R. Stark, Ross J. Keenan, Daniel J. Myall, Michael R. MacAskill, Toni L. Pitcher, Leslie Livingston, Sophie Grenfell, Kyla-Louise Horne, Bob N. Young, Maddie J. Pascoe, Mustafa M. Almuqbel, Jian Wang, Steven H. Marsh, David H. Miller, John C. Dalrymple-Alford, Tim J. Anderson

**Affiliations:** ^1^New Zealand Brain Research Institute, Christchurch, New Zealand; ^2^Department of Medicine, University of Otago, Christchurch, New Zealand; ^3^Brain Research New Zealand Rangahau Roro Aotearoa Centre of Research Excellence, Christchurch, New Zealand; ^4^Pacific Radiology Group, Christchurch, New Zealand; ^5^Department of Neurology, Huashan Hospital, Fudan University, Shanghai, China; ^6^Department of Physics and Astronomy, University of Canterbury, Christchurch, New Zealand; ^7^Institute of Neurology, University College London, London, United Kingdom; ^8^Department of Psychology, University of Canterbury, Christchurch, New Zealand; ^9^Department of Neurology, Christchurch Hospital, Christchurch, New Zealand

**Keywords:** Parkinson's disease, amyloid PET, Florbetaben, dementia, centiloid, mild cognitive impairment

## Abstract

The extent to which Alzheimer neuropathology, particularly the accumulation of misfolded beta-amyloid, contributes to cognitive decline and dementia in Parkinson's disease (PD) is unresolved. Here, we used Florbetaben PET imaging to test for any association between cerebral amyloid deposition and cognitive impairment in PD, in a sample enriched for cases with mild cognitive impairment. This cross-sectional study used Movement Disorders Society level II criteria to classify 115 participants with PD as having normal cognition (PDN, *n* = 23), mild cognitive impairment (PD-MCI, *n* = 76), or dementia (PDD, *n* = 16). We acquired 18F-Florbetaben (FBB) amyloid PET and structural MRI. Amyloid deposition was assessed between the three cognitive groups, and also across the whole sample using continuous measures of both global cognitive status and average performance in memory domain tests. Outcomes were cortical FBB uptake, expressed in centiloids and as standardized uptake value ratios (SUVR) using the Centiloid Project whole cerebellum region as a reference, and regional SUVR measurements. FBB binding was higher in PDD, but this difference did not survive adjustment for the older age of the PDD group. We established a suitable centiloid cut-off for amyloid positivity in Parkinson's disease (31.3), but there was no association of FBB binding with global cognitive or memory scores. The failure to find an association between PET amyloid deposition and cognitive impairment in a moderately large sample, particularly given that it was enriched with PD-MCI patients at risk of dementia, suggests that amyloid pathology is not the primary driver of cognitive impairment and dementia in most patients with PD.

## Introduction

Motor impairment is the cardinal feature of early Parkinson's disease (PD), but progressive cognitive impairment and dementia (PDD) eventually become major debilitating symptoms for patients (1). PDD arises in over 80% of patients (2), leading to substantial caregiver and financial burden, reduced quality of life, early institutionalization and premature death (3). Progression to PDD involves a complex, multisystem brain degeneration (1, 4). Alzheimer's disease (AD) neuropathology, including misfolded beta-amyloid (Aβ), may influence the emergence of PDD by acting synergistically with α-synucleinopathy (4–8). Neuropathological investigations of Aβ suggest an association with cognitive impairment and increased deposition in PDD, at least in a subset of patients (4, 5, 9–11). Similarly, increased concentrations of Aβ in cerebrospinal fluid have been associated with cognitive dysfunction and dementia in PD (12–16), although some studies have not found this relationship (17, 18). While both neuropathological and CSF markers suggest an association with cognitive decline, the cerebral deposition of amyloid is, however, not ubiquitous and the neuropathology underlying the development of PDD remains heterogeneous (19–21).

*In vivo* imaging of α-synuclein is currently not possible, but positron emission tomography (PET) imaging allows an *in vivo* test of an association between amyloid deposits and cognition in PD (22, 23). Amyloid PET imaging, however, has produced conflicting results in PD, especially with respect to cognitive decline. Gomperts and colleagues (22), found no difference in amyloid accumulation in the precuneus between a group of PD patients with mild cognitive impairment (PD-MCI) and cognitively normal patients at baseline, but the baseline presence of amyloid was weakly associated with cognitive decline an average of 2.5 years later, suggesting that amyloid may be a better marker of future rather than current cognitive status in PD. While Fiorenzato et al. (24), suggest a modest association with cognitive decline, other *in vivo* amyloid imaging studies suggest that amyloid deposition may occur in only a minority of PD patients, even in PDD (23, 25–31). However, these previous PET studies have used relatively small samples and the robustness of their findings may be compromised by low statistical power, lack of thorough cognitive characterization, or not accounting for age.

We therefore investigated the relationship between amyloid deposition and cognitive impairment in PD using [^18^F] Florbetaben (FBB) PET imaging in a large, cognitively well-characterized group of PD participants that included cases with normal cognition (PDN), mild cognitive impairment (PD-MCI) and dementia (PDD). Patients meeting PD-MCI criteria are at a 7-fold higher risk of conversion to PDD over a 4-year period compared to patients who do not meet these criteria (32). Thus, the sample was enriched by recruiting a large proportion of PD-MCI patients; this is a group in whom intervention to prevent progression to dementia is particularly pertinent.

Since previous studies have suffered from inconsistent and variable standardization procedures, we used centiloid scaling in the present investigation. The centiloid scale facilitates direct comparison of amyloid deposition across different imaging centers, analysis methods, amyloid ligands (incorporating ^11^C- and ^18^F-based ligands), and diseases (33, 34). This is achieved by appling a linear scaling to amyloid PET data to an average value of zero in high-certainty amyloid-negative subjects, and to an average of 100 in typical AD subjects (33). In this first application of centiloid standardization in PD, we (1) investigated the relationship between amyloid deposition and cognitive impairment in a group of well-characterized PD participants representative of the broad cognitive spectrum, and (2) established the distribution of centiloid values across the cognitive spectrum in PD.

## Materials and Methods

As part of an ongoing longitudinal study, a convenience sample of 118 PD participants meeting the UK Parkinson's Disease Society's criteria for idiopathic PD (35) was recruited from volunteers at the Movement Disorders Clinic at the New Zealand Brain Research Institute, Christchurch, New Zealand. We invited people representative of the broad spectrum of cognitive status in PD to participate, i.e., from normal cognition to dementia, although we particularly encouraged participation from individuals with PD-MCI. Exclusion criteria included atypical Parkinsonian disorders; prior learning disability; previous history of other neurological conditions including moderate-severe head injury, stroke, vascular dementia; and major psychiatric or medical illness in the previous 6 months. Neuroradiological screening (RJK) excluded two participants with multifocal infarcts and one in whom part of the bolus injection extravasated into the soft tissue. Participants completed a neuropsychological battery, MRI scanning session, and [^18^F] Florbetaben (FBB) PET imaging. All participants gave written informed consent, with additional consent from a significant other when appropriate. The study was approved by the regional Ethics Committee of the New Zealand Ministry of Health (No. URB/09/08/037).

### Diagnostic Criteria and Assessment

Comprehensive neuropsychological assessment fulfilling the Movement Disorders Society (MDS) Task Force Level II criteria was used to diagnose PD-MCI (32, 36). Five cognitive domains were examined (executive function; attention, working memory and processing speed; learning and memory; visuospatial/visuoperceptual function; and language; see Supplementary Table 1 for a list of the specific tests) (32). Within each cognitive domain, standardized scores from the constituent neuropsychological tests were averaged to provide individual cognitive domain scores; global cognitive performance for each participant was expressed as an aggregate z score obtained by averaging four domain scores (language domain excluded). PD-MCI cases had unimpaired functional activities of daily living, as verified by interview with a significant other, and scored 1.5 SD or more below normative data on at least two measures within at least one of the five cognitive domains (32). Dementia was defined using MDS criteria as significant cognitive impairments (2 SD below normative data) in at least two of five cognitive domains, plus evidence of significant impairment in everyday functional activities, not attributed to motor impairments (37). Participants also completed the Montreal Cognitive assessment (MoCA). All assessments and scans were performed with no disruption to participants' usual medication regimen. PD participants were classified as either cognitively normal (PDN, *n* = 23), with mild cognitive impairment (PD-MCI; *n* = 76), or with dementia (PDD; *n* = 16). Assessors were blinded to amyloid status.

### PET Acquisition

[^18^F] Florbetaben (FBB) was manufactured in Melbourne, Australia, by Cyclotek Pty Ltd, and transported by air freight to Christchurch, New Zealand, with sufficient radioactivity for three participant doses, despite the passage of three half-lives in transit. After receiving an intravenous injection of 300 MBq ± 20% FBB, participants were scanned in “list mode” on a GE Discovery 690 PET/CT scanner, 90–110 min after injection. Images were reconstructed using an iterative time-of-flight plus SharpIR algorithm. Standardized uptake value (SUV), defined as the decay-corrected brain radioactivity concentration normalized for injected dose and body weight, was calculated at each voxel. A low dose CT scan was acquired immediately prior to PET scanning for attenuation correction. Voxel size in the reconstructed 20 min PET image was 1.2 × 1.2 × 3.2 mm^3^.

### MRI Acquisition

MR images were acquired on a 3T General Electric HDxt scanner (GE Healthcare, Waukesha, USA) with an eight-channel head coil. A volumetric T1-weighted (inversion-prepared spoiled gradient recalled echo (SPGR), TE/TR = 2.8/6.6 ms, TI = 400 ms, flip angle = 15 deg, acquisition matrix = 256 × 256 × 170, FOV = 250 mm, slice thickness = 1 mm) was acquired to facilitate spatial normalization of FBB PET images. Additional T2-weighted and T2-weighted fluid-attenuated inversion recovery (FLAIR) images were acquired to enable a clinical read.

### Classification of FBB Images

Visual classification of FBB scans as positive or negative is accurate and reliable for detection of cases with histology-defined plaques (38). A neuroradiologist (RJK, with both in-person and e-training), blinded to cognitive status, rated each scan as amyloid-positive or -negative. That judgment was based on the assessment of FBB uptake in gray vs. white matter in the lateral temporal, frontal, posterior cingulate/precuneus, and parietal lobes (in accordance with the NeuraCeq™ guidelines: https://www.accessdata.fda.gov/drugsatfda_docs/label/2014/204677s000lbl.pdf).

An additional approach using standardized uptake value ratios (SUVR) or centiloids (see below) was also used to categorize FBB scans. An ROC analysis [using the R package “pROC” (39)] was used to identify the optimum centiloid cut-off to separate positive and negative scans.

### Image Processing

#### MRI

CAT12 (r934, http://www.neuro.uni-jena.de/cat/), a toolbox of SPM12 (v6685, http://www.fil.ion.ucl.ac.uk/spm/), running in Matlab 9.0.0 (R2016a), was used to process T1-weighted structural images. Images were bias corrected, spatially normalized via DARTEL (using the MNI-registered template provided within CAT12), modulated to compensate for the effect of spatial normalization, and classified into gray matter (GM), white matter (WM), and cerebrospinal fluid (CSF), all within the same generative model (40).

### PET Data

FBB PET images were coregistered to each person's T1-weighted image and warped into MNI space using the MRI-derived deformation fields. We then created a standardized uptake value ratio (SUVR) image in each individual by scaling to the mean radioactivity in the Centiloid project whole cerebellum reference region of interest. Mean cortical SUVR was extracted from the standard centiloid cortical region. Lastly, SUVR images were smoothed using an 8 mm isotropic Gaussian kernel for whole-brain analysis.

### Centiloid Calibration

We performed a level 3 centiloid calibration (Supplementary Material) to verify agreement between the standard centiloid processing method (which utilized SPM8) and our processing method (which utilized CAT12 normalization) (33, 34). All calibration parameters were within expected values, validating our processing methods (slope = 0.998, intercept = −0.187, and *R*^2^ = 0.995). Cortical centiloid values were calculated in all PD participants using the FBB-to-centiloid conversion equation (centiloid units = 153.4 × SUVR_FBB_ – 154.9) (34).

### Regions of Interest (ROIs)

While our principal analysis focused on cortical Aβ deposition, a number of both pathological and imaging studies suggest a potential relationship between Aβ accumulation in the striatum, thalamus, and globus pallidus and cognitive decline (10, 24, 41–43). We therefore specifically investigated *a priori* ROIs, including the caudate, putamen, thalamus, globus pallidus, and precuneus. The precuneus was included as a representative cortical region that exhibits very high amyloid load in AD (44). As standard centiloid regions do not exist for these structures, we calculated average SUVR within these regions defined by the Harvard-Oxford cortical and subcortical atlases in MNI152 space (45–48).

### Statistical Analysis

Bayesian models were fitted using the “brms” (v2.2.0) package (49) in R (v3.4.4). In each model, four chains with 2,000 iterations each were used to generate the posterior sample. Model comparison using LOOIC (leave-one-out information criterion) was performed when models included correlated predictors or predictive performance was being evaluated (50). A lower LOOIC score, by at least twice the standard error of the estimated difference, indicated a model with a better fit, and consequently whether a specific predictor significantly improved model fit. Baseline demographic and neuropsychological group differences were analyzed using linear models (in brms). Analysis code and data are available at https://osf.io/5fqb9/.

#### Region of Interest (ROI) Analysis

We investigated the relationship between FBB uptake and cognition in PD using Bayesian regression models including age and sex.

1. We first tested for evidence of varying cortical amyloid deposition (centiloid) across the cognitive subgroups (PDN, PD-MCI, PDD).
2. We aimed to predict a continuous measure of global cognitive ability (aggregate cognitive z score) as a function of age, sex, and cortical FBB binding (centiloid). We evaluated the importance of predictors by model comparison, using LOOIC. That is, we compared a model predicting global cognitive ability with and without cortical FBB binding in order to determine whether cortical FBB binding improved prediction of global cognitive ability. This same procedure was repeated for the memory domain score.
3. Lastly, regional SUVR from the *a priori* ROIs was modeled as a function of age, age-by-ROI, sex, and global cognitive ability-by-ROI interaction, in order to investigate the relationship between FBB uptake and cognition in the different ROIs.

#### Whole-Brain Voxel-Wise Analysis (SUVR)

We used a standard, frequentist ANCOVA model (with age and sex as covariates) to assess the spatial distribution of amyloid deposition across cognitive subgroups (we specifically investigated the contrasts: PDD > PD-MCI, PDD > PDN, and PD-MCI > PDN). In addition, we ran three multiple linear regression models to investigate the association between voxel-wise FBB SUVR and continuous measures of (1) global cognitive ability (cognitive z score), (2) memory domain score, and (3) age. Age and sex were included as covariates in the global cognitive ability and memory domain models; only sex was included in the age model. Voxel-wise comparisons were performed using a gray matter mask and a permutation-based inference tool for non-parametric thresholding [“randomise” (51) in FSLv5.0.9]. For each contrast, the null distribution was generated from 5,000 permutations and the alpha level set at *p* < 0.05, corrected for multiple comparisons [family-wise error correction using threshold-free cluster-enhancement (TFCE)].

## Results

Table 1 summarizes the demographic and clinical information for PD participants. Twenty-one of 115 (18%) had positive FBB scans on visual assessment. We identified a centiloid cut-off of 31.3 (equivalent SUVR = 1.21), which yielded sensitivity (to visually assessed positive scans) = 100%, specificity = 92.6%, and AUC [95% confidence interval] = 0.98 [0.97, 1.0]. We also identified a significant association between centiloid and age (*r* = 0.011 [0.005, 0.017] SUVR/year, or 9.3% per decade).

**Table 1:** Demographic, cognitive, and clinical metrics.

	**PDN**	**PD-MCI**	**PDD**	**Linear model**
*n*	23	76	16	–
Female, No. [%]	8 (35)	18 (24)	3 (19)	–
Age, years	70 (6)	72 (6)	77 (6)	PDD > PDN & PD-MCI
Education, years	12 (2)	13 (3)	12 (2)	~
PD symptom duration, years	7.4 (5)	7.3 (4)	8.5 (5)	~
MoCA	26 (2)	23 (3)	16 (5)	PDN > PD-MCI > PDD
Cognitive Z score	0.28 (0.48)	−0.81 (0.53)	−1.89(0.57)^a^	PDN > PD-MCI > PDD
Memory domain score	0.52 (0.86)	−0.82 (0.85)	−1.82(0.67)^a^	PDN > PD-MCI > PDD
Dose, MBq	294 (20)	300 (16)	290 (27)	~
Aβ positive, No. [%]^b^	4 [17]	11 [14]	6 [38]	–
Mean cortical SUVR_NS_	1.11 (0.13)	1.12 (0.18)	1.28 (0.30)	PDD > PDN & PD-MCI
Mean cortical CL	16 (19)	18 (27)	42 (44)	PDD > PDN & PD-MCI

Values are mean (standard deviation) unless specified;

a *Cognitive z scores and memory domain scores for seven PDD participants were imputed from restricted neuropsychological data due to their inability to complete the full cognitive assessment*.

b *Visual assessment of amyloid positive/negative reported. ~, no evidence of a difference; –, no statistical test applicable or was not performed. Pairwise group estimates were considered different if 95% uncertainty intervals did not overlap. MBq, megabecquerel; MoCA, Montreal Cognitive Assessment; Aβ, Amyloid beta; SUVR_NS_, Standardized uptake value ratio with “non-standard” processing (see Supplementary Material), CL, centiloid*.

### Regional Amyloid Distribution in PD

**Figure 1:**
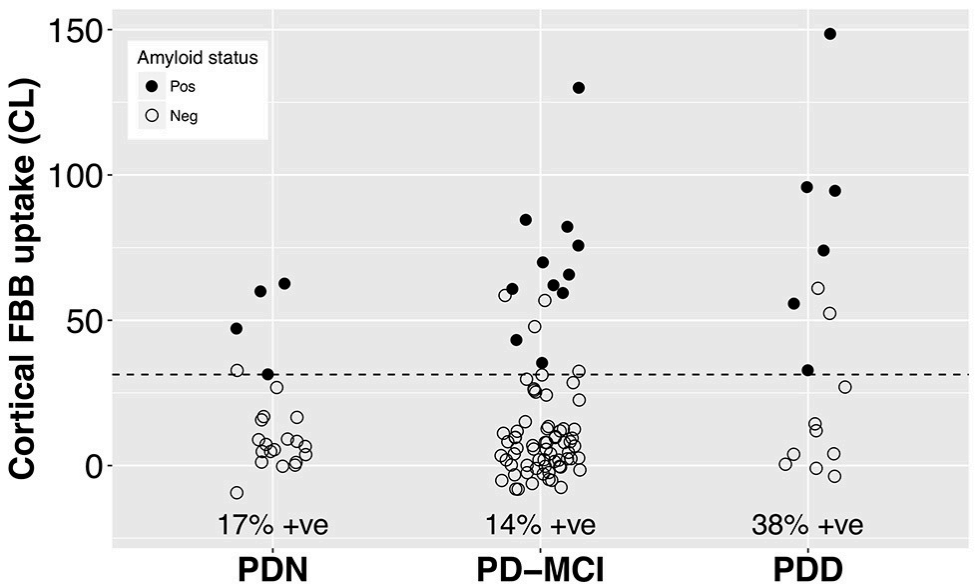
Cortical FBB uptake by cognitive group. We found evidence of increased cortical amyloid accumulation in PDD relative to PDN and PD-MCI, however this was explained by the older age of the PDD group (Table 1). The dashed line at CL = 31.3 indicates the ROC-defined optimal centiloid cut-off in this sample, with sensitivity to clinically positive cases = 100%, specificity = 92.6%, AUC [95% confidence interval] = 0.98 [0.97, 1.0]. FBB, Florbetaben; PDN, Parkinson's with normal cognition; PD-MCI, Parkinson's with mild cognitive impairment; PDD, Parkinson's with dementia; CL, centiloid.

**Figure 2:**
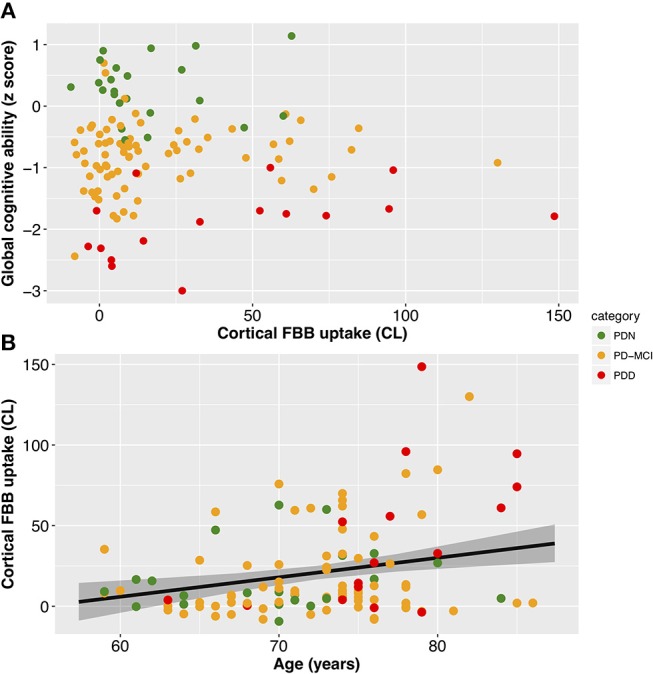
Associations between cortical amyloid deposition and global cognitive ability and age. **(A)** Scatter plot showing no evidence of a significant association between global cognitive ability (Cognitive z score) and cortical amyloid (CL; Table 1). **(B)** Scatter plot of cortical amyloid (CL) vs. age (years). FBB uptake was associated with age (slope = 1.5 CL/year, 95% uncertainty interval [0.6, 2.3], equivalent to SUVR of 0.011/year [0.005, 0.017]). Black line depicts estimate from the Bayesian model fit and the shaded area indicates the 95% credible interval. Color represents cognitive status: green—Parkinson's with normal cognition (PDN), orange—Parkinson's with mild cognitive impairment (PD-MCI), red—Parkinson's with dementia (PDD). CL, centiloid.

**Figure 3:**
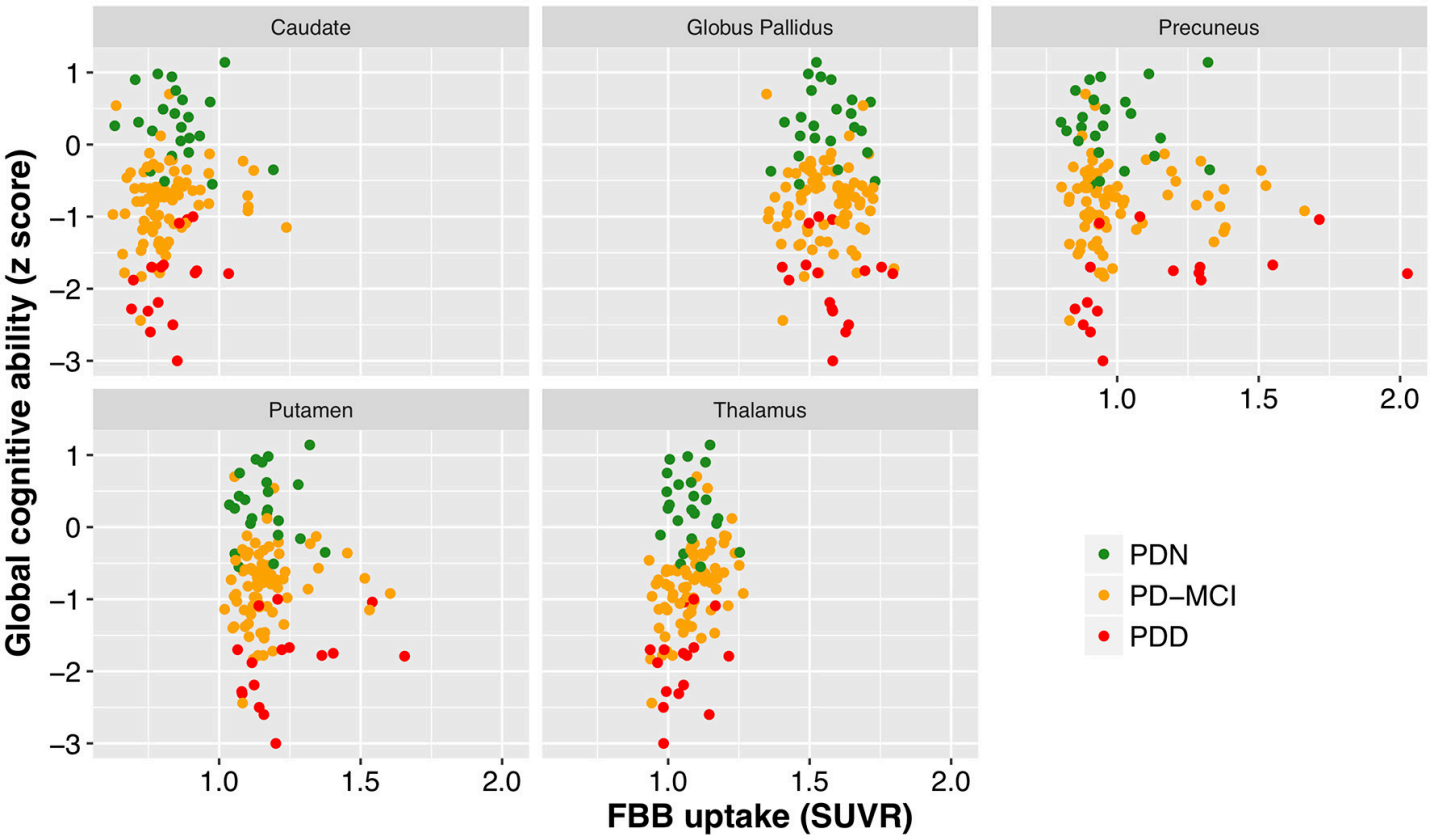
Cognitive performance as a function of mean standardized uptake volume ratios (Florbetaben) within a number of brain regions. While different regions exhibited different levels of amyloid deposition, there was a clear lack of relationship between cognitive performance (cognitive z score) and SUVR within all of the regions examined. FBB, Florbetaben; SUVR, standardized uptake volume ratio. Color represents cognitive status: green—Parkinson's with normal cognition (PDN), orange—Parkinson's with mild cognitive impairment (PD-MCI), red—Parkinson's with dementia (PDD).

With a simple model that only considered the discrete cognitive groups, we found evidence of increased cortical amyloid accumulation in PDD relative to PDN and PD-MCI (Figure 1; Table 1). However, adding age as a covariate to the model and using LOOIC to compare models, showed that age, rather than cognitive group, was predictive of increased cortical amyloid accumulation (Figure 2B; Supplementary Material). When attempting to predict cognition from cortical amyloid deposition, the addition of FBB uptake (centiloid) to a model resulted in marginally worse out-of-sample prediction of global cognitive score [LOOIC (standard error) = 1.8 (0.8), Figure 2A] and memory score [0.7 (2.1), data not shown] than simpler models, which only included age and an intercept. This indicates FBB uptake has little, if any, relationship with cognitive impairment in our PD sample. In *a priori* ROIs, including age and sex, we saw no evidence of association between FBB uptake (SUVR) and either global cognitive or memory score (Figure 3).

### Whole-Brain Voxel-Wise Amyloid Distribution in PD

**Figure 4:**
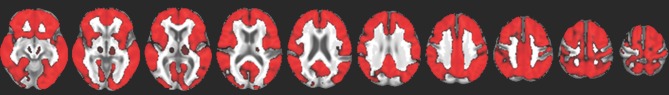
Red indicates voxels with a significant positive association between FBB uptake and age (TFCE-corrected *p* < 0.05), overlaid on a study-specific structural image. This association was evident throughout the cortex and in the thalamus but not in the striatum.

We identified no evidence of a difference in amyloid deposition across PD cognitive groups (TFCE-corrected, *p* < 0.05). Furthermore, we identified no evidence of association between SUVR and either global cognitive ability or memory domain scores. There was, however, a widespread positive association between SUVR and age over the cortex (Figure 4).

## Discussion

Using FBB PET imaging in 115 PD patients across the cognitive spectrum, we observed significantly higher cortical amyloid accumulation in our PDD group relative to other cognitive subgroups, but model comparison indicated this was due to the older age of the PDD group.

Visual assessment revealed amyloid positive proportions of 17, 14, and 38% in PDN, PD-MCI, and PDD groups, respectively. The prevalence of amyloid positivity reported in the literature is variable, ranging from 0 to 53% in PDN (26, 27, 30, 31, 52), 0–47% in PD-MCI (23, 26, 27, 30, 31, 52), and an estimated point prevalence of 34% in PDD (23). Nevertheless, these proportions of amyloid positivity across the cognitive spectrum in PD are substantially lower than levels seen in Alzheimer's dementia (88%) (53) or amnestic MCI (69%) (54), and are closer to levels seen in elderly controls (11.6% at age 60, 23.8% at 70, and 34.5% at 80 years) (53). The association we observed between amyloid deposition and age (*r* = 0.011 [0.005, 0.017] SUVR/year, or 9.3% per decade) is similar to that reported in the healthy elderly population (^11^C-PiB uptake increased at 0.016 SUVR/year, ~10% per decade) (54), indicating that a PD-specific influence on amyloid accumulation is unlikely. Although global SUVR measures obtained from PiB and FBB PET in the same subjects have excellent linear correlation, the above rates are not directly comparable as different reference regions were used to define SUVR (for example, we used the whole cerebellum while Villemagne et al. (54), used the cerebellar cortex). Nevertheless, amyloid load in our PD sample appears to be consistent with levels seen in the general elderly population, as well as previous PD studies (2, 23, 31), and any increases in our PDD group can be explained by their older age. Not accounting for age may help explain the recent report of association between amyloid deposition and global cognition in a subset of the Parkinson's Progression Marker Initiative (24).

Ideally we would have used a predefined centiloid threshold derived from a large population study to define amyloid positivity in our PD sample. However, to the best of our knowledge, this is not currently possible. SUVR cut-off values are well established, but recent work demonstrates that specific thresholds are, as expected, highly dependent on the reference regions and processing methodology (7, 55). Therefore, a threshold derived using a particular method should not necessarily be applied to a different processing methodology, even after centiloid standardization (55). Many potential thresholds are available: a phase III FBB study identified a histopathologically-confirmed amyloid positivity cut-off of SUVR = 1.478 (56); Jack et al. (57), report a Pittsburgh Compound B-derived cut-off of SUVR = 1.42 and CL = 19; Bullich et al. (58), reported FBB thresholds using cerebellar cortex (SUVR = 1.43) and non-centiloid whole cerebellum (SUVR = 0.96) as reference regions. However, it would be inappropriate to apply these cut points to our current dataset as image processing and reference regions differed from the standard centiloid SUVR method. Su et al. (55), presented a centiloid cut-off using standard reference regions (CL = 6.8) based on an ROC analysis to classify young, amyloid negative participants from AD patients in the GAAIN dataset. This surprisingly low threshold may be driven by differences in non-specific binding and tracer delivery differences between young and old participants. In any case, standardized centiloid analyses of large cohorts are needed to establish appropriate centiloid thresholds, which will lead to greater applicability of the centiloid scale.

In this study, we used a well-validated visual assessment to clinically rate scans as being amyloid positive or negative (38). As there is not an accepted threshold based on standardized centiloid reference regions, we defined an amyloid positivity centiloid cut-off threshold in our sample. Our cut-off (CL = 31.3, SUVR = 1.21) corresponds well to the estimated value proposed by Rowe and colleagues (34) in the context of AD (CL = 25–30), however our estimated threshold may be biased by the low number of Aβ positive patients.

Our results suggest a lower prevalence of amyloid-positive PDD individuals than in dementia with Lewy bodies (DLB); neither of these two conditions exhibit the proportions of amyloid-positive cases reported in Alzheimer's dementia (23, 30, 59, 60). While some have reported an association between cognitive ability and cortical SUVR in DLB (28), the largest study (including the most thoroughly profiled group of DLB to date) did not find an association between amyloid deposition and clinical profile, despite showing increased amyloid accumulation vs. controls (59). We confirm here a similar lack of association in PD between amyloid deposition and cognitive impairment, with age explaining the increased FBB-uptake observed in our PDD group.

Most of our PD patients were within the normal centiloid range (comparted to control data from the Global Alzheimer's Association Information Network used for level 3 centiloid standardization: http://www.gaain.org/centiloid-project), with few showing AD-like levels of cortical amyloid. Hence Aβ pathology is unlikely to be a dominant causal factor in the majority of individuals with PD or PDD.

PET measures of amyloid do not suggest plaques as a primary pathology for dementia in PD, but amyloid may play a part in conjunction with other pathologies, such as alpha-synuclein and hyper-phosphorylated tau. It is expected that tau deposition will correlate more directly with current cognitive ability, due to its association with accelerating neuronal injury. Initial tau PET imaging in PD and DLB demonstrates a spectrum of deposition, with reports of both association (30, 52) and lack of association (26, 61) with cognitive impairments in PD. Thus, consideration of amyloid, tau, and alpha-synuclein deposition in the same individuals may ultimately provide a more complete description of how pathological processes potentially interact to affect cognition in PD. A potential scenario for prediction of future outcomes will most likely synthesize an array of biomarkers representative of these and other pathologies (21, 62).

Our results suggest that amyloid deposition is neither necessary nor sufficient to explain cognitive decline and dementia in PD. The current study cannot address the role that amyloid accumulation plays in AD, but it does raise the question as to the fundamental relationship between amyloid plaques and dementia. While the amyloid cascade hypothesis remains the leading candidate to explain the pathophysiology of AD, it not universally accepted (63, 64). Amyloid beta may be a downstream result, and not necessarily the cause, of AD (65).

Limitations of this study include the absence of a healthy control group. Analyses were restricted to the effects of varying levels of cognitive impairment within PD. All comparisons to healthy controls were based on comparable reports from the literature. However, the primary aim of this work was to investigate the relationship between amyloid deposition and cognitive impairment *within* a group of well-characterized PD participants. Even when following level II criteria for PD-MCI, considerable variability exists across those diagnosed as PD-MCI; some exhibit single domain and others multi-domain impairment (32). It is possible that different subtypes may exhibit greater or lesser underlying Aβ. Nonetheless, Aβ was not associated with global cognitive ability or memory function. We do not know the APOE genotype of our participants, which has been shown to correlate with amyloid deposition (31, 54). We also did not have histopathological confirmation of amyloid plaque accumulation, although recent work demonstrates tight agreement between visual assessment of amyloid PET and histopathological evidence in AD (58). Lastly, recent work suggests that partial volume correction can improve the ability of FBB PET to discriminate between AD patients and healthy controls (66). We did not perform this step because partial volume correction methods are still highly variable across centers, with no consensus on optimal methods, and have not been incorporated into centiloid standardization procedures yet (33).

In this cross-sectional investigation of a large, cognitively well-characterized PD group, we found increased cortical amyloid accumulation in PDD, but this was explained by the older age of the PDD group. We found no associations between amyloid load and continuous measures of cognitive performance. This suggests that Aβ accumulation is not the primary cause of cognitive impairments in PD. Low levels of amyloid may, however, still interact synergistically with other PD pathological processes, thereby accelerating other pathways to dementia.

## Data Availability

Analysis code and data are available on the Open Science Framework at https://osf.io/5fqb9/.

## Ethics Statement

All participants gave written consent, with additional consent from a significant other when appropriate. The study was approved by the regional Ethics Committee of the New Zealand Ministry of Health (No. URB/09/08/037).

## Author Contributions

TM, DJM, MM, TP, DHM, JD-A, and TA conceptualized and designed the study. TM, MS, and DJM drafted the manuscript, performed the image processing, and statistical analyses. TM, MM, DHM, RK, DJM, LL, JD-A, and TA obtained funding for the study. RK, LL, DJM, MM, TP, and SM provided administrative, technical, and material support. All authors contributed to acquisition, analysis, or interpretation of data. All authors contributed to manuscript revision, read, and approved the submitted version.

### Conflict of Interest Statement

DHM has received honoraria through payments to UCL Institute of Neurology for Advisory Committee and/or Consultancy advice from Novartis and Mitsubishi Pharma Europe, and grants through payments to UCL Institute of Neurology from Biogen Idec and Novartis. The remaining authors declare that the research was conducted in the absence of any commercial or financial relationships that could be construed as a potential conflict of interest.
