# Supplementary material for: Beta Amyloid Deposition Is Not Associated With Cognitive Impairment in Parkinson's Disease
Source: Front Neurol. 2019 Apr 24;10:391. doi: 10.3389/fneur.2019.00391 (PMC6492461; doi:10.3389/fneur.2019.00391)
Supplement: Supplementary file 1 [file Data_Sheet_1.docx]

Supplementary Material

# Supplementary Data

**1.1 Centiloid calibration (level 3):** We performed a level 3 centiloid (CL) calibration to verify agreement between the standard CL processing method (which utilized SPM8) and our ‘non-standard’ processing method ([1](#_ENREF_1), [2](#_ENREF_2)), which utilized CAT12 normalization.

FBB and structural MR images in 25 elderly subjects and 10 young controls,([2](#_ENREF_2)) and standard CL regions (whole cerebellum and cortex), were downloaded from the Global Alzheimer’s Association Information Network (GAAIN: <http://www.gaain.org>). We then created cortical SUVR values using our ‘non-standard’ CAT12 normalization methodology for all FBB images, following steps outlined in ‘Image processing’ of the main manuscript. We then compared our ‘non-standard’ cortical SUVR values to the published ‘standard’ cortical SUVR values available from the GAAIN images (using a linear model, per Klunk et al. ([1](#_ENREF_1))). We demonstrated that our ‘non-standard’ processing pipeline “does not introduce errors into the data” ([1](#_ENREF_1)). A scatter plot is displayed in Supplementary Figure 1, showing the relationship and linear equation linking SUVR_NS_ (our processing) and SUVR_STD_ (standard processing ([1](#_ENREF_1), [2](#_ENREF_2))). This equation was then used to convert our ‘non-standard’ cortical FBB SUVR values to ‘standard’ cortical FBB SUVR values; these ‘standard’ cortical SUVR values were then converted into CL units using the FBB-to-CL conversion equation([2](#_ENREF_2)) (CL units = 153.4 × SUVR_FBB_ − 154.9). We then plotted calculated CL values obtained from our ‘non-standard’ processing against the published CL values (Supplementary Figure 2). The expectation is that the slope will be between 0.98 and 1.02 (actual: 1.000), the intercept will be between -2 and 2 CL (actual: -0.0357), and R^2^>0.98 (actual: 0.995). All parameters were well within the expected values for level 3 calibration of a non-standard method; hence, CL values reported in the current manuscript meet all specifications and can therefore be interpreted as standardized, quantitative measure of amyloid deposition.


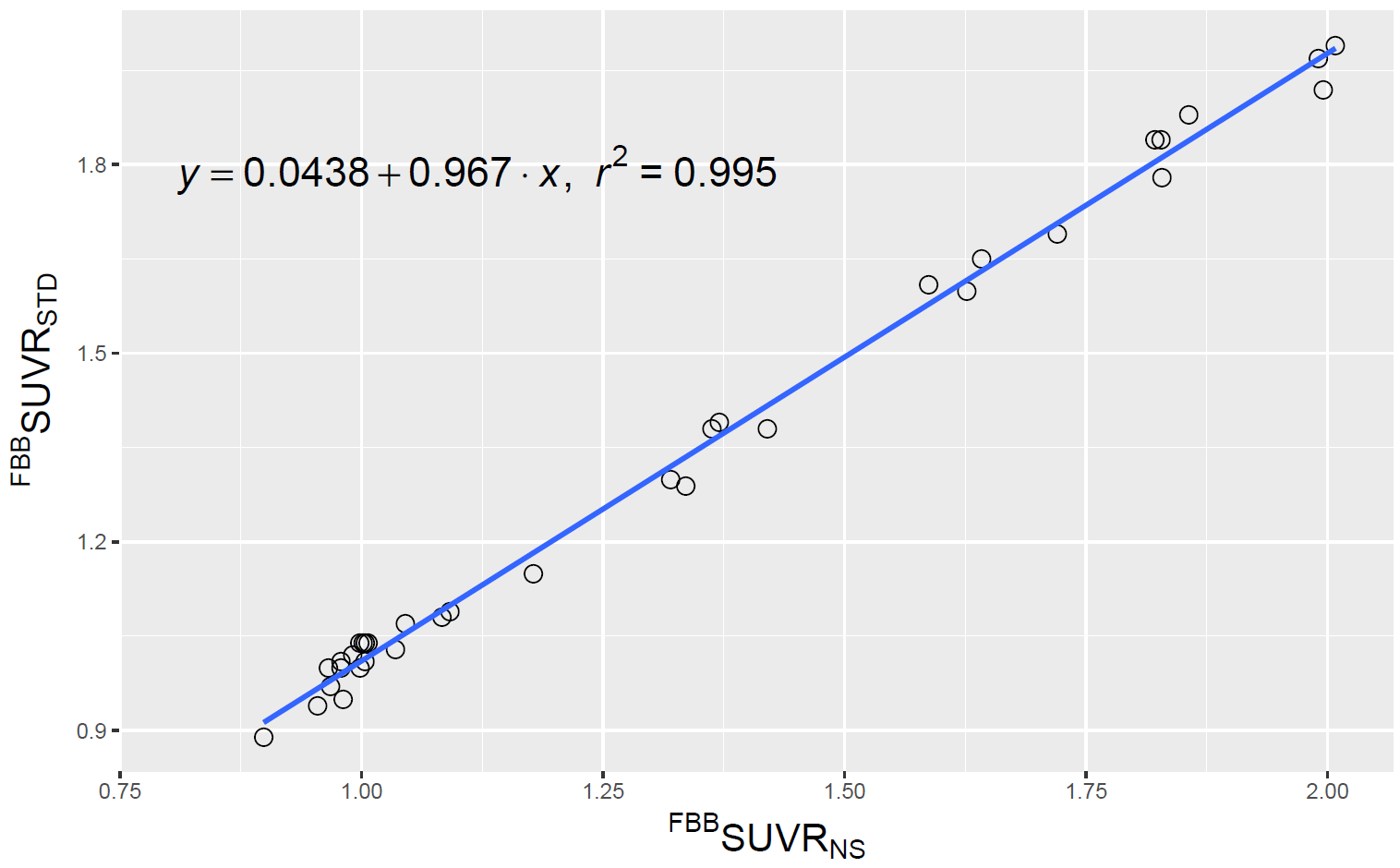
**Supplementary Figure 1:** Scatterplot of published ‘standard’ cortical FBB SUVR values (^FBB^SUVR_STD_) versus our ‘non-standard’ cortical FBB SUVR values (^FBB^SUVR_NS_) using CAT12 normalization and from 25 elderly subjects and 10 younger controls described by Rowe and colleagues, ([2](#_ENREF_2)) downloaded from the GAAIN website. SUVR values were created using the Centiloid Project whole cerebellum reference region and cortical region. The calculated linear equation was used to correct our ‘non-standard’ SUVR values to the ‘standard method’ described by Klunk et. al. ([1](#_ENREF_1)).


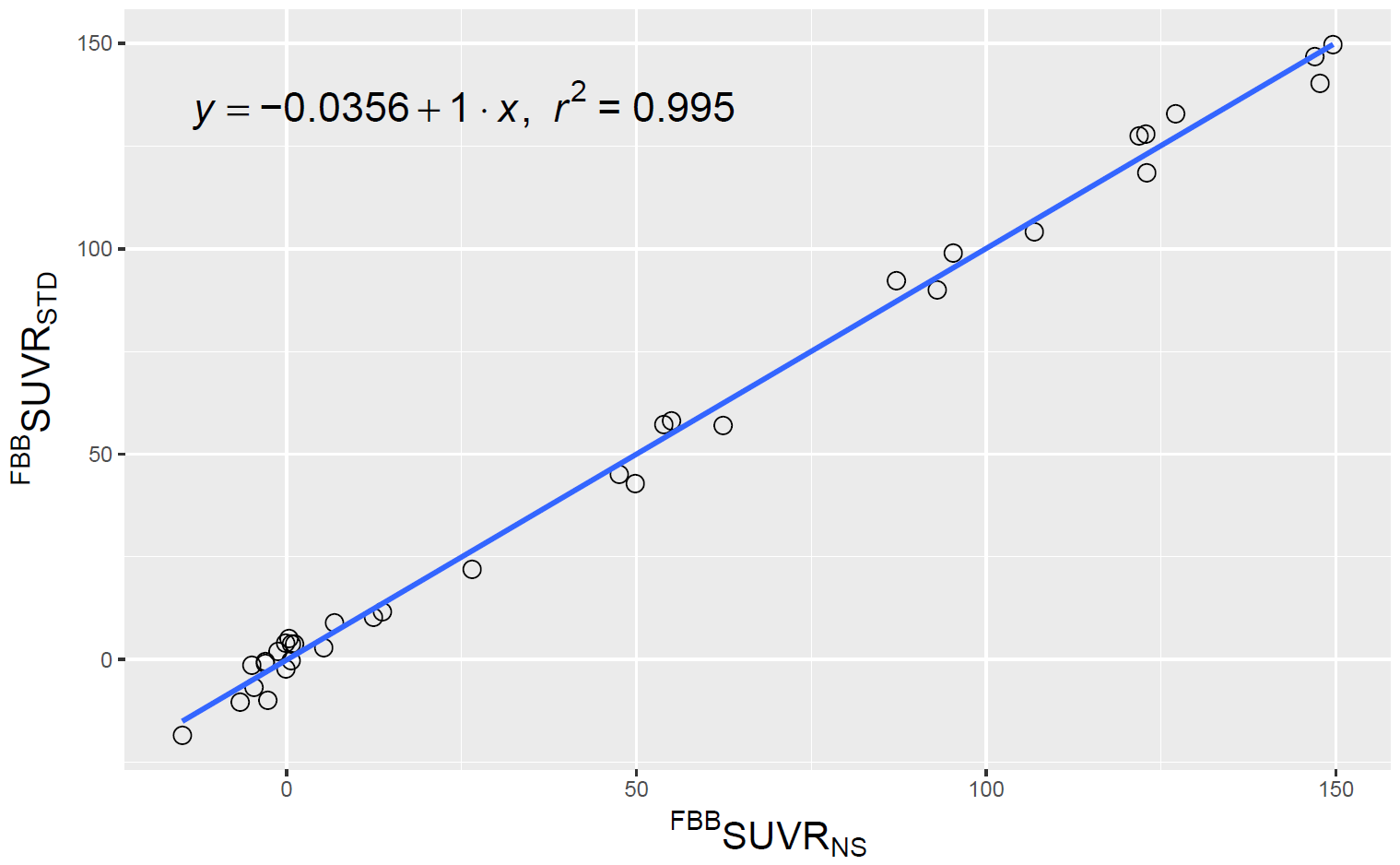


**Supplementary Figure 2:** Scatterplot of the published CL values from the ‘standard’ processing (^FBB^CL_STD_) versus our calculated CL values (^FBB^CL_NS_) from the GAAIN dataset ([2](#_ENREF_2)). The plot shows excellent agreement between the two processing streams.

**1.2 Cognitive category and age: Model comparison**

As cognitive status and age are correlated (PDD are on average older than PD-MCI who are on average older than PD-N), care is required in interpreting a model that includes both of these predictors. Comparing models using LOOIC allows the determination of relative predictive information contained in variables. Adding cognitive category to a model with only an intercept resulted in a decrease in LOOIC of 5.5 +/- 7.0 (a smaller LOOIC value indicates a better model fit). In comparison, adding age to a model with only an intercept gave a decrease in LOOIC of 10.6 +/- 6.0. Adding cognitive category to a model which included age resulted in a small decrease of 1.5 +/- 5.3. The effects of age and cognitive category are small effects on the LOOIC scale, indicating that neither has a large predictive ability. However, these results indicate that cognitive category has minimal predictive value once age is known, suggesting that age is more likely the stronger predictor.

| **Supplementary Table 1:** The five cognitive domains and individual tests | | | | |
| --- | --- | --- | --- | --- |
| **Attention, working memory and processing speed** | **Executive function** | **Visuoperceptual/**  **visuospatial** | **Learning and memory** | **Language** |
| Digits forward/backward | Action (verb) fluency | Judgment of line orientation | CVLT-II SF acquisition | Boston naming test |
| Digit ordering | Letter fluency  (D-KEFS) | Fragmented letters | CVLT-II SF short delay (30 secs)^a^ | DRS-2 similarities sub-tests |
| Map test (test of everyday attention) | Category fluency  (D-KEFS) | Rey complex figure copy | CVLT-II SF long delay (10 mins)^a^ | ADAS-Cog (object and finger naming, commands, comprehension, spoken language and word finding difficulties) |
| Stroop color reading | Category switching  (D-KEFS) | Picture completion | RCF test – short delay (3 mins)^b^ |  |
| Stroop word reading | Trails B |  | RCF test – long delay (30 mins)^b^ |  |
| Trails A | Stroop interference  (D-KEFS) |  |  |  |
| ^a, b^ An impairment in either or both delay components of these episodic memory tests were counted as one impairment.  ADAS-Cog, Alzheimer’s Dementia Assessment Scale; CVLT-II SF, California Verbal Language Test-II Short Form; D-KEFS, Delis-Kaplan executive function system; DRS-2; Dementia Rating Scale-2, RCF test; Rey Complex Figure Test. | | | | |

**Supplementary References**

1. Klunk WE, Koeppe RA, Price JC, Benzinger TL, Devous MD, Sr., Jagust WJ, et al. The Centiloid Project: standardizing quantitative amyloid plaque estimation by PET. *Alzheimers Dement* (2015) 11(1):1-15. Epub 2014/12/03. doi: 10.1016/j.jalz.2014.07.003. PubMed PMID: 25443857; PubMed Central PMCID: PMC4300247.

2. Rowe CC, Doré V, Jones G, Baxendale D, Mulligan RS, Bullich S, et al. 18F-Florbetaben PET beta-amyloid binding expressed in Centiloids. *Eur J Nucl Med Mol Imaging* (2017) 44(12):2053-9. doi: 10.1007/s00259-017-3749-6.
